# Diagnostic Accuracy and Fine-Grained Image-Interpretation Performance of Multimodal Large Language Models on Bilingual Radiology Questions: Cross-Sectional Benchmark Study

**DOI:** 10.2196/98849

**Published:** 2026-11-11

**Authors:** Yijie Liu, Xin Zhang, Jia Xu, Manxin Huang, Yinting Xiong, Wenqian Wu

**Affiliations:** 1 Union Hospital, Department of Ultrasound Medicine, Tongji Medical College, Huazhong University of Science and Technology, 1277 Jiefang Avenue, Wuhan, 430022, China., Wuhan, Hubei, China, 86 27-85726430, 86 27-85726430; 2 Hubei Province Clinical Research Center for Medical Imaging, Wuhan, Hubei, China; 3 Hubei Province Key Laboratory of Molecular Imaging, Wuhan, Hubei, China

**Keywords:** large language models, radiology, medical imaging, multiple-choice questions, AI, benchmarking

## Abstract

**Background:**

Multimodal large language models (LLMs) are rapidly expanding in radiology and digital health; however, their diagnostic performance across diverse clinical scenarios and fine-grained visual reasoning tasks requires systematic evaluation.

**Objective:**

The study aims to evaluate the diagnostic accuracy and fine-grained visual reasoning performance of 5 multimodal LLMs with bilingual medical imaging questions.

**Methods:**

This cross-sectional benchmark study evaluated bilingual medical imaging questions using zero-shot prompting on 5 LLMs: GPT-5.2, Gemini 3 Pro, Doubao, Yuanbao, and Llama 4 Maverick. Subgroup analyses by language (English/Chinese), question type (image/text), imaging modality (computed tomography, radiography, magnetic resonance imaging, ultrasound, and nuclear medicine), and anatomical system (neurologic, respiratory, cardiovascular, digestive, genitourinary, and musculoskeletal) evaluated intermodel accuracy and intramodel consistency. Image-based visual reasoning performance was assessed across modality, anatomy, lesion detection, and localization.

**Results:**

A total of 298 questions (198 image-based, 100 text-based) were evaluated. Gemini 3 Pro achieved the highest overall accuracy (n=268, 89.9%, 95% CI 86.0%‐92.9%), significantly outperforming Doubao (n=235, 78.9%; *P*<.001), GPT-5.2 (n=233, 78.2%; *P*<.001), Yuanbao (n=216, 72.5%; *P*<.001), and Llama 4 Maverick (n=216, 72.5%; *P*<.001). All models performed better on text-based than image-based questions (Yuanbao, *P*<.001; Doubao, *P*=.02; GPT-5.2, *P*<.001; Gemini 3 Pro, *P*=.002; Llama 4 Maverick, *P*<.001). GPT-5.2 (*P*<.001), Llama 4 Maverick (*P*=.03), and Yuanbao (*P*=.03) favored English questions, while intramodel performance remained stable across modalities (Yuanbao, *P*=.46; Doubao, *P*=.21; GPT-5.2, *P*=.26; Gemini 3 Pro, *P*=.96; Llama 4 Maverick, *P*=.45) and anatomical systems (Yuanbao, *P*=.82; Doubao, *P*=.52; GPT-5.2, *P*=.35; Gemini 3 Pro, *P*=.13; Llama 4 Maverick, *P*=.97). Despite near-perfect macroscopic visual recognition (modality and anatomy: 99.0%‐100.0%), accuracy dropped in complex tasks. Gemini 3 Pro outperformed Yuanbao (*P*<.001), GPT-5.2 (*P*=.002), and Llama 4 Maverick (*P*<.001) in lesion detection (175/198, 88.4%) and outperformed Yuanbao (*P*<.001), Doubao (*P*=.004), and Llama 4 Maverick (*P*=.004) in lesion localization (189/198, 95.5%).

**Conclusions:**

Gemini 3 Pro demonstrated optimal, comprehensive performance and cross-lingual stability. However, current multimodal LLMs face a technical bottleneck in complex visual interpretation—particularly in fine-grained lesion detection and localization—emphasizing the need to prioritize local feature perception in future model development.

## Introduction

In recent years, large language models (LLMs) have provided unprecedented opportunities in radiology [1]. By leveraging robust natural language processing, LLMs have demonstrated immense potential in handling complex radiological text tasks, such as generating structured reports and extracting clinical information [2]. As LLMs evolve toward multimodal architectures, their applications are expanding from singular text interpretation to direct image analysis. However, this cross-modal transition faces significant challenges. While large models exhibit high-level reasoning when processing structured radiological text—capable of generating accurate differential diagnoses solely from textual histories [3,4]—their diagnostic accuracy often declines when presented directly with complex medical images. Two radiology studies evaluating GPT-4–family multimodal systems reported lower performance under image-based conditions than under text-based or text-rich conditions, indicating limitations in radiologic image interpretation for these systems under the settings evaluated [5,6]. This necessitates systematic testing to ascertain the true extent of their visual feature extraction and diagnostic proficiency.

Currently, global LLMs exhibit distinct developmental trajectories in multimodal visual recognition. Globally applied models, such as GPT-5.2 and Gemini 3 Pro, represent the forefront of this technology. The OpenAI GPT series has evolved from early external visual modules to native omni-modal architectures, achieving significant leaps in multimodal feature alignment [7]. The Gemini series, designed natively multimodal from its foundation, leverages long-context reasoning and cross-modal feature fusion to establish a leading position [8]. Concurrently, LLMs developed in China have made rapid breakthroughs. Doubao (ByteDance), a consumer-facing AI assistant powered by ByteDance’s Seed model family, has undergone deep visual cognitive enhancement tailored to the Chinese medical ecosystem, demonstrating high evolutionary efficiency [9]. Notably, Yuanbao (Tencent), a consumer-facing AI assistant evaluated with the DeepSeek-R1 option selected in this study, innovatively integrates the breakthrough DeepSeek-R1 architecture. By using large-scale reinforcement learning and chain-of-thought deep reasoning, it substantially enhances the model’s capacity for self-reflection in complex medical diagnosis [10,11]. Cross-platform comparisons between these emerging regional models and global general models help explore the impact of diverse training data ecosystems on model efficacy, providing valuable empirical references for evaluating their generalizability [12,13]. However, despite these architectural advancements, the diagnostic stability and visual recognition capabilities of these multimodal LLMs across diverse medical imaging modalities (eg, computed tomography [CT], magnetic resonance imaging [MRI], radiography, ultrasound, and nuclear medicine [NM]) remain largely unclear, highlighting a critical gap that necessitates systematic evaluation. The combined evaluation of multimodal LLMs developed by companies based in China and the United States within the same standardized bilingual benchmark, together with structured image-interpretation assessment across imaging modalities and anatomical systems, remains limited.

Medical multiple-choice questions (MCQs), encompassing both formal board-style examinations and clinical image challenges, serve as reliable tools for evaluating the clinical reasoning of large models [14-16]. Therefore, we used MCQs that covered bilingual contexts, multiple imaging modalities, and various anatomical systems with zero-shot prompting [14]. The purpose of this cross-sectional benchmark study was to compare the capabilities of different multimodal LLMs (Gemini 3 Pro, Doubao, GPT-5.2, Yuanbao, and Llama 4 Maverick) in medical image recognition and clinical diagnostic reasoning and to systematically analyze influencing factors. In addition to comprehensively analyzing the impact of these variables on overall accuracy, we uniquely deconstructed the visual reasoning process of image-based questions into 4 fine-grained dimensions: modality recognition, anatomical localization, lesion detection, and lesion localization. Through this approach, we aimed to accurately delineate the capability boundaries of current multimodal LLMs in radiology, providing solid empirical evidence for future targeted optimization.

## Methods

### Data Collection and Organization

This cross-sectional benchmark study collected a dataset comprising 298 MCQs in radiology, covering both Chinese and English contexts. The Chinese MCQs, which included both text- and image-based items, were sourced from the Chinese National Health Professional Technical Qualification Examinations (Associate Senior Radiology, Nuclear Medicine, and Ultrasound Medicine). To ensure comprehensive modality coverage, the English MCQs were obtained from publicly available educational sources, including the New England Journal of Medicine Image Challenge, BMJ Emergency Medicine Journal Image Challenge, Asian Nuclear Medicine Board sample questions, and Radiopaedia MCQs [15]. The question sources, including question IDs, and identifiable publication periods are summarized in Table S1 in Multimedia Appendix 1. Before model evaluation, all questions and images were reviewed to confirm the absence of direct patient identifiers, and no identifiable patient information was entered into the evaluated LLMs.

Two board-certified radiologists, each with more than 5 years of clinical experience, independently screened and reviewed all questions. The inclusion criteria were as follows: (1) a complete question stem, (2) at least four answer options with a single definitive correct answer, and (3) for image-based questions, a medical image deemed clear and diagnostic by 2 radiologists, derived from a single imaging modality. The exclusion criteria were as follows: (1) nonradiologic images (eg, gross pathology specimens), (2) questions containing video or dynamic images, and (3) mixed-modality questions containing 2 or more imaging modalities within the same item.

The questions finally included were categorized as text-based or image-based. All questions were labeled according to imaging modality (CT, radiography, MRI, ultrasound, and NM) and anatomical system (neurologic, respiratory, cardiovascular, digestive, genitourinary, and musculoskeletal; Figure 1).

**Figure 1.**
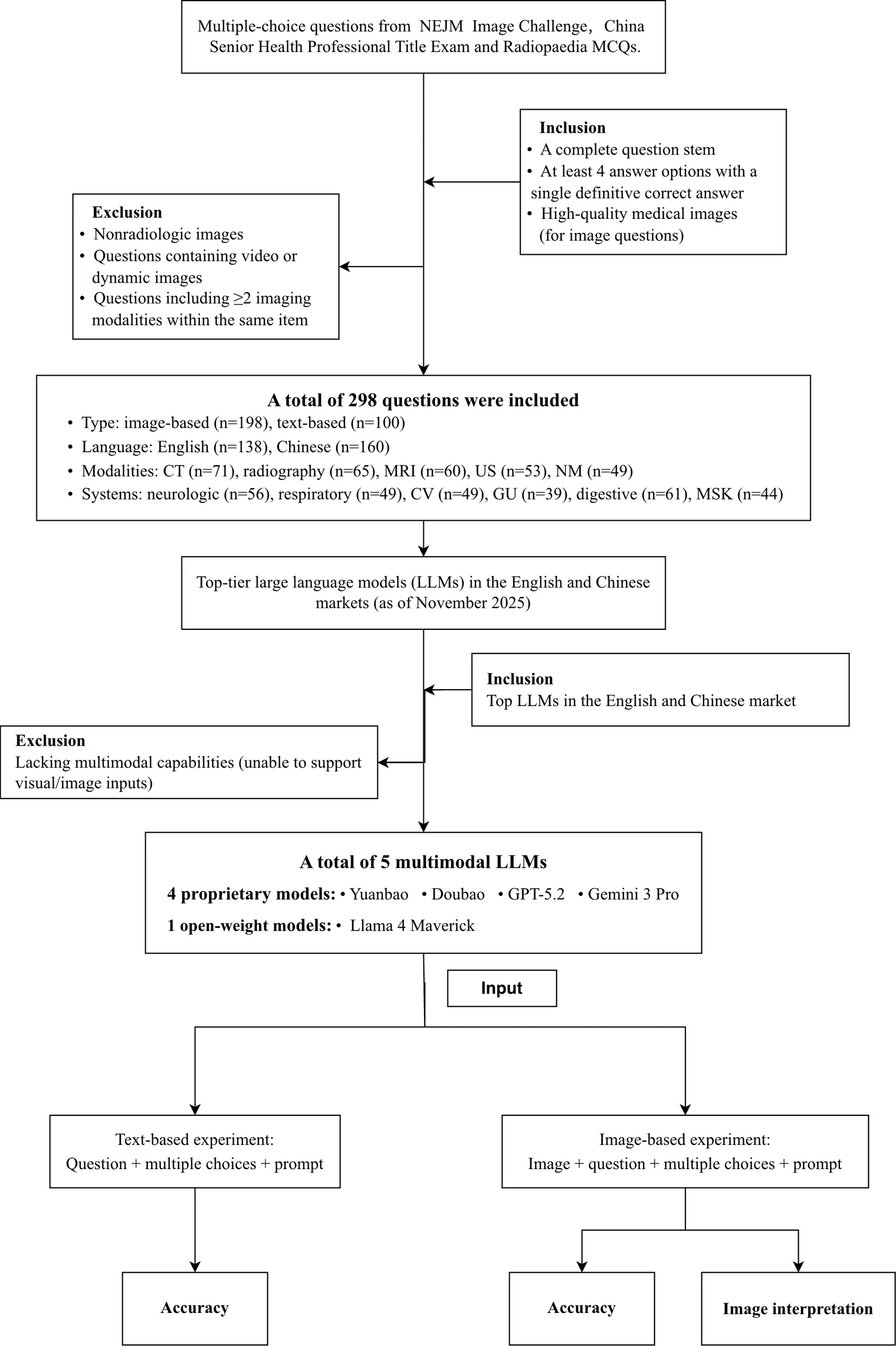
Flow diagram illustrating question selection, large language model (LLM) selection, and workflow. CT: computed tomography, CV: cardiovascular; GU: genitourinary; MCQ: multiple-choice question; MRI: magnetic resonance imaging; MSK: musculoskeletal; NEJM: *New England Journal of Medicine*; NM: nuclear medicine.

### Ethical Considerations

This study used only publicly available examination questions and published educational materials without identifiable patient information. Under applicable national ethical review regulations in China [16], an ethics review was not required for this study; accordingly, no formal exemption determination, protocol number, and application number were issued.

### Model Selection and Evaluation

Four proprietary multimodal LLMs were selected based on their visual processing capabilities and leading market positions as of November 2025: Gemini 3 Pro (Google), Doubao (ByteDance), GPT-5.2 (OpenAI), and Yuanbao (Tencent; with the DeepSeek-R1 option selected). Models lacking visual input support were excluded. The 4 proprietary multimodal LLMs were evaluated between December 14, 2025, and January 31, 2026, via the official web-based interfaces of each model, without API access [17]. Detailed model configurations and access conditions are provided in Table S2 in Multimedia Appendix 1. The user-facing reasoning settings were Auto mode for GPT-5.2, Pro mode for Gemini 3 Pro, Deep Thinking for Doubao, and DeepSeek-R1 with Deep Thinking for Yuanbao. To provide an open-weight reproducibility anchor alongside the 4 proprietary systems, a separate evaluation of Meta Llama 4 Maverick 17B 128E Instruct was conducted in September 2026 using the same 298 questions. The model was accessed through a dedicated fixed end point, provided by Together AI, with detailed deployment and inference configurations provided in Table S3 in Multimedia Appendix 1. To prevent contextual carryover and potential memory bias, each question was evaluated independently; a new conversation was initiated for each question in the 4 web-based systems, whereas Llama 4 Maverick was submitted as an independent API request. Default model parameters were maintained for the 4 web-based systems to simulate real-world clinical workflows, and a zero-shot prompting strategy was applied [18]. Zero-shot prompting was selected because our objective was to evaluate the baseline, out-of-the-box performance of the evaluated models without task-specific demonstrations or prompt optimization. Input configurations were standardized by question type. For text-based questions, the input comprised the complete stem, answer options, and task instructions. For image-based questions, the medical image was uploaded alongside the text. To minimize translation-induced bias and simulate authentic clinical scenarios, prompts were constructed in the original language of the question (Chinese or English; eg, “Please select the correct answer for the above question”; Figure 2).

**Figure 2.**
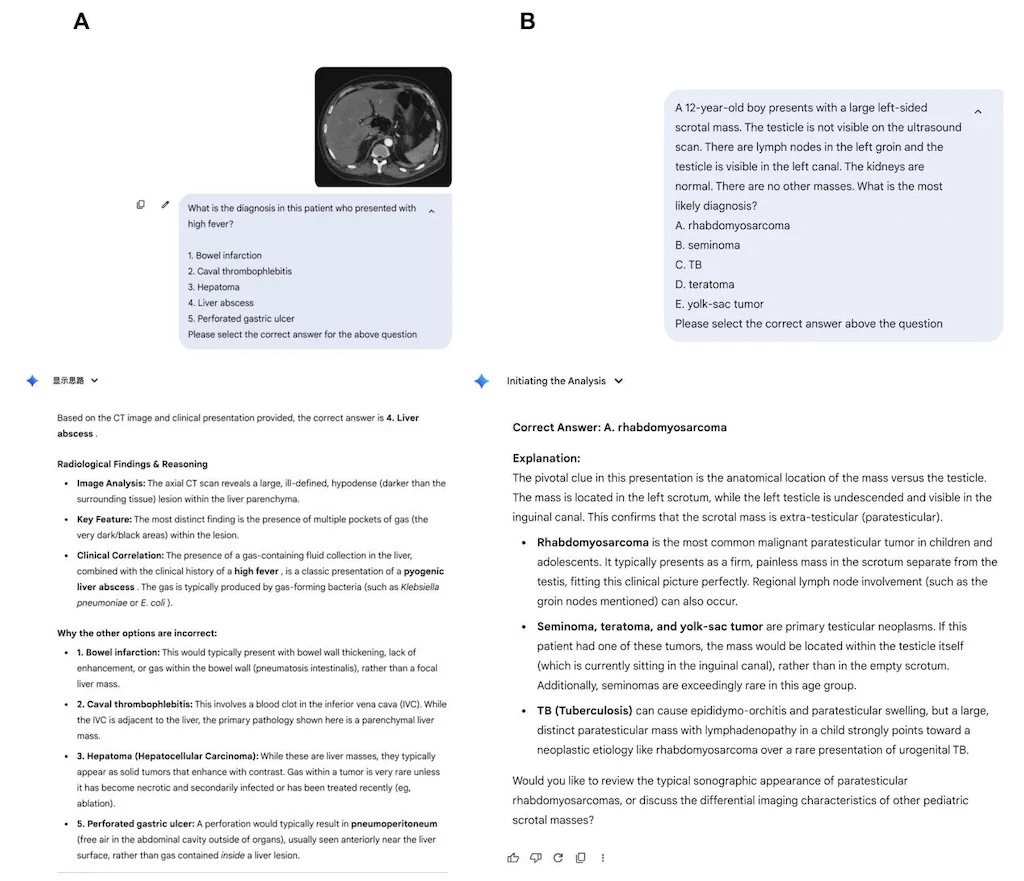
Representative examples of multimodal large language models (LLMs) answering multiple-choice questions in medical imaging. (A) Screenshot shows Gemini 3 Pro (Google) correctly answering an English image-based question. (B) Screenshot shows Gemini 3 Pro (Google) correctly answering an English text-based question. CT: computed tomography.

The primary evaluation metric was the binary accuracy (correct or incorrect) of the model’s final answer, defined as the proportion of questions for which the model-selected option matched the reference answer. For image-based questions, to further investigate visual reasoning performance, 2 radiologists jointly reviewed the generated reasoning content. They reached a consensus judgment for each outcome, and only the final consensus result was recorded for analysis. Scoring was performed across four binary dimensions: (1) modality (whether the model correctly identified the imaging technique, such as distinguishing CT from MRI), (2) anatomy (whether the model correctly identified the depicted anatomical region or organ), (3) lesion detection (whether the model explicitly identified the abnormality in the reasoning text), and (4) localization (whether the model correctly described the precise location of the lesion) [19]. To verify the reliability and reproducibility of the 4 image-interpretation outcomes, 2 radiologists independently and blindly evaluated the diagnostic outputs generated by 5 LLMs for 40 randomly selected image-based questions (approximately 20% of the 198 image-based questions). The 40 questions ×5 LLMs yielded 200 LLM responses. For each response, both physicians determined whether the output was correct or incorrect across 4 prespecified dimensions: modality recognition, anatomical recognition, lesion detection, and lesion localization. Agreement was assessed separately for each dimension, with 200 paired ratings per dimension. Both radiologists were blinded to each other’s ratings.

### Statistical Analysis

For intermodel comparisons, the Cochran *Q* test was used to evaluate overall differences in accuracy among the 5 models. Accuracy estimates were reported with 95% Wilson CIs. Analyses were conducted on the overall dataset and stratified by language, question type (image-based vs text-based), imaging modality, and anatomical system. Given the influence of visual information on model reasoning, separate analyses based on modality and anatomical system were performed within the image- and text-based subgroups. If the global test indicated statistical significance, post hoc pairwise comparisons were conducted using the 2-sided exact McNemar test [20]. Pairwise effect sizes were reported as absolute differences in accuracy with 95% Newcombe CIs for paired proportions. For intramodel consistency, the chi-square test was used to assess the performance stability of each model across independent subgroups (eg, Chinese vs English questions). Yates continuity correction was applied to 2×2 comparisons of language and question type, whereas standard Pearson chi-square tests were used for comparisons across the 5 imaging modalities and 6 anatomical systems. The 4 image-interpretation outcomes were evaluated for image-based questions. Interrater reliability for the 4 image-interpretation outcomes was assessed using standard Cohen κ. To account for highly skewed marginal distributions in certain outcomes, prevalence-adjusted and bias-adjusted kappa (PABAK) was also calculated. The 95% CIs were computed for all point estimates. The strength of agreement was categorized according to the Landis and Koch criteria: <0.00, poor; 0.00‐0.20, slight; 0.21‐0.40, fair; 0.41‐0.60, moderate; 0.61‐0.80, substantial; and 0.81‐1.00, almost perfect. To control for type I error inflation from multiple pairwise comparisons, all post hoc *P* values were corrected using the Holm-Bonferroni method. All 298 questions received valid responses from all 5 models, with no missing, refused, excluded, or imputed observations. All statistical tests were 2-sided, and a *P* value of less than .05 was considered indicative of a statistically significant difference, with Holm-adjusted *P* values used for post hoc pairwise comparisons. Data analysis and figure generation were performed using Python software (version 3.13).

## Results

### Characteristics of MCQs

A total of 298 medical imaging questions were included. The dataset comprised 198 (66.4%) image-based questions and 100 (33.6%) text-based questions. In terms of language distribution, the dataset was balanced between Chinese (n=160, 53.7%) and English (n=138, 46.3%). Questions covered multiple imaging modalities with relatively even distribution: CT (n=71, 23.8%), radiography (n=65, 21.8%), MRI (n=60, 20.1%), ultrasound (n=53, 17.8%), and NM (n=49, 16.4%). Questions were distributed across 6 major anatomical systems to ensure comprehensive subspecialty coverage: digestive (n=61, 20.5%), neurologic (n=56, 18.8%), cardiovascular (n=49, 16.4%), respiratory (n=49, 16.4%), musculoskeletal (n=44, 14.8%), and genitourinary (n=39, 13.1%; Table 1).

**Table 1.** Characteristics of all selected questions (N=298).

Characteristic	Number of questions, n (%)
Question type	
Image-based	198 (66.4)
Text-based	100 (33.6)
Language	
Chinese	160 (53.7)
English	138 (46.3)
Imaging modality	
CT^a^	71 (23.8)
Radiography	65 (21.8)
MRI^b^	60 (20.1)
Ultrasound	53 (17.8)
NM^c^	49 (16.4)
Anatomical system	
Neurologic	56 (18.8)
Digestive	61 (20.5)
Cardiovascular	49 (16.4)
Respiratory	49 (16.4)
Musculoskeletal	44 (14.8)
Genitourinary	39 (13.1)
Question source	
Chinese National Health Professional Technical Qualification Examinations	160 (53.7)
New England Journal of Medicine Image Challenge	71 (23.8)
Radiopaedia MCQs^d^	54 (18.1)
BMJ Emergency Medicine Journal Image Challenge	7 (2.4)
Asian Nuclear Medicine Board sample questions	6 (2.0)

a CT: computed tomography.

b MRI: magnetic resonance imaging.

c NM: nuclear medicine.

d MCQ: multiple-choice question.

### Overall Accuracy and Stratified Analysis by Language and Question Type

Across the entire dataset, the overall accuracy of the 5 evaluated multimodal LLMs ranged from 72.5% (n=216) to 89.9% (n=268). Overall accuracy differed among the 5 LLMs (Cochran *Q* test, *P<*.001). Pairwise comparisons demonstrated that Gemini 3 Pro achieved higher overall accuracy (n=268, 89.9%, 95% CI 86.0%‐92.9%) than Doubao (n=235, 78.9%, 95% CI 73.9%‐83.1%; *P<*.001), GPT-5.2 (n=233, 78.2%, 95% CI 73.2%‐82.5%; *P<*.001), Yuanbao (n=216, 72.5%, 95% CI 67.2%‐77.2%; *P<*.001), and Llama 4 Maverick (n=216, 72.5%, 95% CI 67.2%‐77.2%; *P<*.001). No significant differences were detected among Doubao, GPT-5.2, Yuanbao, and Llama 4 Maverick (Table 2 and Figure 3A). Pairwise absolute accuracy differences with 95% Newcombe CIs are reported in Table S4 in Multimedia Appendix 1.

**Table 2.** Comparison of accuracy among large language models across different subgroups^a^.

Subgroup	N	Yuanbao, accuracy (95% Wilson score CI)	Doubao, accuracy (95% Wilson score CI)	GPT-5.2, accuracy (95% Wilson score CI)	Gemini 3 Pro, accuracy (95% Wilson score CI)	Llama 4 Maverick, accuracy (95% Wilson score CI)	*P* value
Language							
Chinese	160	66.9 (59.3‐73.7)	76.9 (69.8‐82.7)	68.1 (60.6‐74.8)	86.9 (80.8‐91.3)^b^^,c,d^	66.9 (59.3‐73.7)	<.001
English	138	79.0 (71.4‐85.0)	81.2 (73.8‐86.8)	89.9 (83.7‐93.9)^b^	93.5 (88.1‐96.5)^b^^,^^d^^,^^e^	79.0 (71.4‐85.0)	<.001
Type							
Image-based	198	63.6 (56.7‐70.0)	74.7 (68.3‐80.3)^e^	71.7 (65.1‐77.5)^b^	85.9 (80.3‐90.0)^b^^,^^c^^,^^d^^,^^e^	66.2 (59.3‐72.4)	<.001
Text-based	100	90.0 (82.6‐94.5)	87.0 (79.0‐92.2)	91.0 (83.8‐95.2)	98.0 (93.0‐99.4)^d^^,^^e^	85.0 (76.7‐90.7)	.002
Total	298	72.5 (67.2‐77.2)	78.9 (73.9‐83.1)	78.2 (73.2‐82.5)	89.9 (86.0‐92.9)^b^^,^^c^^,^^d^^,^^e^	72.5 (67.2‐77.2)	<.001

a Data are presented as accuracy (%) with 95% Wilson score CIs in parentheses. The *P* value in the last column indicates the global difference among the 5 models calculated using the Cochran *Q *test.

b Significant in comparison with Yuanbao, *P*<.05.

c Significant in comparison with GPT-5.2, *P*<.05.

d Significant in comparison with Llama 4 Maverick, *P*<.05 (Holm-Bonferroni corrected).

e Significant in comparison with Doubao, *P*<.05.

**Figure 3.**
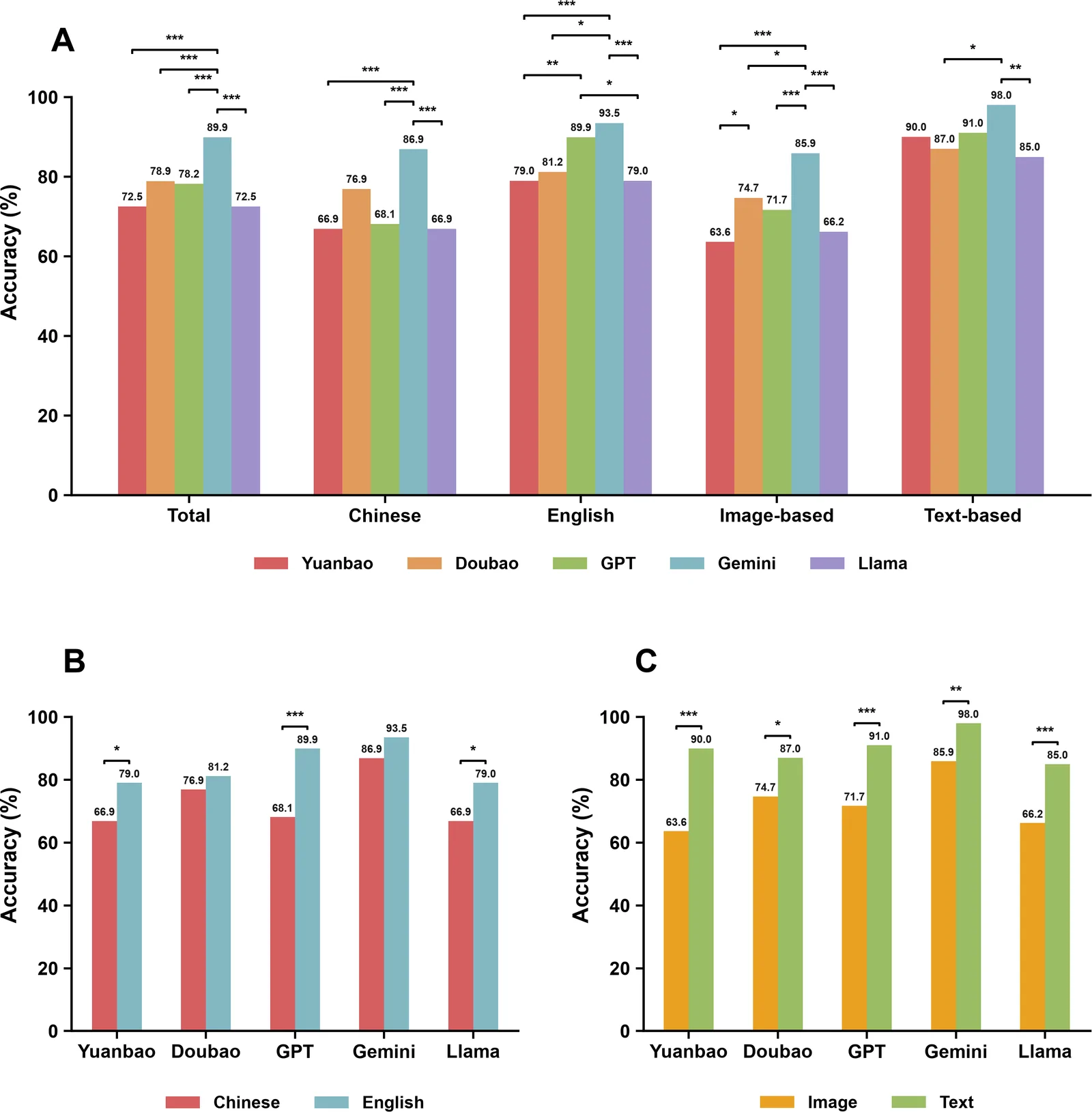
Intermodel and intramodel accuracy comparison of large language models (LLMs) on medical imaging questions. (A) The bar graph shows the performance of 5 LLMs—Yuanbao, Doubao, GPT (GPT-5.2), Gemini (Gemini 3 Pro), and Llama (Llama 4 Maverick)—across 5 key subsets of the dataset: the total dataset, Chinese-language questions, English-language questions, image-based questions, and text-based questions. (B) The bar graph details the within-model (intramodel) performance comparison between Chinese and English questions. (C) The bar graph details the within-model performance comparison between image-based and text-based questions. Significant differences are indicated by brackets and asterisks: *P<.05, **P<.01, ***P<.001.

For Chinese-language (n=160) questions, overall differences were observed among models (*P<*.001). Gemini 3 Pro (n=139, 86.9%, 95% CI 80.8%‐91.3%) outperformed GPT-5.2 (n=109, 68.1%, 95% CI 60.6%‐74.8%; *P<*.001), Yuanbao (n=107, 66.9%, 95% CI 59.3%‐73.7%; *P<*.001), and Llama 4 Maverick (n=107, 66.9%, 95% CI 59.3%‐73.7%; *P<*.001). The difference between Doubao (n=123, 76.9%, 95% CI 69.8%‐82.7%) and Gemini 3 Pro was not statistically significant. For English-language questions (n=138), overall differences were also observed (*P<*.001). Gemini 3 Pro (n=129, 93.5%, 95% CI 88.1%‐96.5%) achieved higher accuracy than Doubao (n=112, 81.2%, 95% CI 73.8%‐86.8%; *P=*.01), Yuanbao (n=109, 79.0%, 95% CI 71.4%‐85.0%; *P<*.001), and Llama 4 Maverick (n=109, 79.0%, 95% CI 71.4%‐85.0%; *P<*.001). GPT-5.2 (n=124, 89.9%, 95% CI 83.7%‐93.9%) also outperformed Yuanbao (*P=*.006) and Llama 4 Maverick (*P=*.01). No significant differences were detected between Gemini 3 Pro and GPT-5.2 (Table 2 and Figure 3A).

Regarding intramodel language comparisons, Yuanbao, Llama 4 Maverick, and GPT-5.2 achieved higher accuracy on English questions than on Chinese questions (*P=*.03, *P=*.03, and *P<*.001, respectively). No significant difference was detected for Doubao or Gemini 3 Pro (Figure 3B).

For image-based (n=198) questions, overall differences were observed among models (*P<*.001). Gemini 3 Pro (n=170, 85.9%, 95% CI 80.3%‐90.0%) outperformed Doubao (n=148, 74.7%, 95% CI 68.3%‐80.3%; *P=*.02), GPT-5.2 (n=142, 71.7%, 95% CI 65.1%‐77.5%; *P<*.001), Yuanbao (n=126, 63.6%, 95% CI 56.7%‐70.0%; *P<*.001), and Llama 4 Maverick (n=131, 66.2%, 95% CI 59.3%‐72.4%; *P<*.001). Doubao also outperformed Yuanbao (*P=*.02). For text-based (n=100) questions, overall differences were observed (*P=*.002). Pairwise comparisons showed that Gemini 3 Pro (n=98, 98.0%, 95% CI 93.0%‐99.4%) achieved higher accuracy than Doubao (n=87, 87.0%, 95% CI 79.0%‐92.2%; *P=*.03) and Llama 4 Maverick (n=85, 85.0%, 95% CI 76.7%‐90.7%; *P=*.002; Table 2 and Figure 3A).

Within-model comparisons showed that all 5 models achieved higher accuracy on text-based questions than on image-based questions (Yuanbao, *P*<.001; Doubao, *P*=.02; GPT-5.2, *P*<.001; Gemini 3 Pro, *P*=.002; Llama 4 Maverick, *P*<.001; Figure 3C).

### Accuracy Analysis Based on Imaging Modality

In the overall dataset stratified by imaging modalities, model accuracy ranged from 66.2% to 91.7%. Overall differences among the 5 models were observed for CT (*P<*.001), MRI (*P=*.03), ultrasound (*P=*.01), and radiography (*P<*.001) questions. No significant overall difference was detected for NM (*P=*.08). Post hoc pairwise comparisons demonstrated that for CT, both Gemini 3 Pro (63/71, 88.7%, 95% CI 79.3%‐94.2%) and GPT-5.2 (58/71, 81.7%, 95% CI 71.2%‐89.0%) achieved higher accuracy than Yuanbao (47/71, 66.2%, 95% CI 54.6%‐76.1%; *P=*.001 and *P=*.009, respectively); Gemini 3 Pro also outperformed Llama 4 Maverick (49/71, 69.0%, 95% CI 57.5%‐78.6%; *P=*.02). For MRI, although the overall difference was significant (*P=*.03), no pairwise differences remained significant after Holm-Bonferroni correction. For ultrasound, Gemini 3 Pro (48/53, 90.6%, 95% CI 79.7%‐95.9%) achieved higher accuracy than GPT-5.2 (38/53, 71.7%, 95% CI 58.4%‐82.0%; *P=*.02) and Llama 4 Maverick (36/53, 67.9%, 95% CI 54.5%‐78.9%; *P=*.04). For radiography, Gemini 3 Pro (59/65, 90.8%, 95% CI 81.3%‐95.7%) and Doubao (58/65, 89.2%, 95% CI 79.4%‐94.7%) achieved higher accuracy than Llama 4 Maverick (45/65, 69.2%, 95% CI 57.2%‐79.1%; *P=*.01 and *P=*.02, respectively; Table 3 and Figure 4A).

**Table 3.** Accuracy of large language models according to imaging modality^a^.

Modality	N	Yuanbao, accuracy (95% Wilson score CI)	Doubao, accuracy (95% Wilson score CI)	GPT-5.2, accuracy (95% Wilson score CI)	Gemini 3 Pro, accuracy (95% Wilson score CI)	Llama 4 Maverick, accuracy (95% Wilson score CI)	*P* value
Total							
CT^b^	71	66.2 (54.6‐76.1)	74.6 (63.4‐83.3)	81.7 (71.2‐89.0)	88.7 (79.3‐94.2)^c^^,^^d^	69.0 (57.5‐78.6)	<.001
Radiography	65	75.4 (63.7‐84.2)	89.2 (79.4‐94.7)	86.2 (75.7‐92.5)	90.8 (81.3‐95.7)^d^	69.2 (57.2‐79.1)	<.001
MRI^e^	60	80.0 (68.2‐88.2)	78.3 (66.4‐86.9)	75.0 (62.8‐84.2)	91.7 (81.9‐96.4)	80.0 (68.2‐88.2)	.03
Ultrasound	53	69.8 (56.5‐80.5)	77.4 (64.5‐86.5)	71.7 (58.4‐82.0)	90.6 (79.7‐95.9)^d^^,^^f^	67.9 (54.5‐78.9)	.01
NM^g^	49	71.4 (57.6‐82.2)	73.5 (59.7‐83.8)	73.5 (59.7‐83.8)	87.8 (75.8‐94.3)	77.6 (64.1‐87.0)	.08
Image-based							
CT	51	56.9 (43.3‐69.5)	68.6 (55.0‐79.7)	78.4 (65.4‐87.5)	86.3 (74.3‐93.2)^c^	66.7 (53.0‐78.0)	<.001
Radiography	44	65.9 (51.1‐78.1)	86.4 (73.3‐93.6)	79.5 (65.5‐88.8)	86.4 (73.3‐93.6)^d^	59.1 (44.4‐72.3)	<.001
MRI	40	77.5 (62.5‐87.7)	77.5 (62.5‐87.7)	72.5 (57.2‐83.9)	87.5 (73.9‐94.5)	77.5 (62.5‐87.7)	.31
Ultrasound	33	60.6 (43.7‐75.3)	78.8 (62.2‐89.3)	60.6 (43.7‐75.3)	84.8 (69.1‐93.3)	60.6 (43.7‐75.3)	.02
NM	30	56.7 (39.2‐72.6)	60.0 (42.3‐75.4)	60.0 (42.3‐75.4)	83.3 (66.4‐92.7)	66.7 (48.8‐80.8)	.06
Text-based							
CT	20	90.0 (69.9‐97.2)	90.0 (69.9‐97.2)	90.0 (69.9‐97.2)	95.0 (76.4‐99.1)	75.0 (53.1‐88.8)	.28
Radiography	21	95.2 (77.3‐99.2)	95.2 (77.3‐99.2)	100.0 (84.5‐100.0)	100.0 (84.5‐100.0)	90.5 (71.1‐97.3)	.23
MRI	20	85.0 (64.0‐94.8)	80.0 (58.4‐91.9)	80.0 (58.4‐91.9)	100.0 (83.9‐100.0)	85.0 (64.0‐94.8)	.13
Ultrasound	20	85.0 (64.0‐94.8)	75.0 (53.1‐88.8)	90.0 (69.9‐97.2)	100.0 (83.9‐100.0)	80.0 (58.4‐91.9)	.08
NM	19	94.7 (75.4‐99.1)	94.7 (75.4‐99.1)	94.7 (75.4‐99.1)	94.7 (75.4‐99.1)	94.7 (75.4‐99.1)	>.99

a Data are presented as accuracy (%) with 95% Wilson score CIs in parentheses. The *P* value in the last column indicates the global difference among the 5 models calculated using the Cochran Q test.

b CT: computed tomography.

c Significant in comparison with Yuanbao, *P<*.05.

d Significant in comparison with Llama 4 Maverick, *P<*.05 (Holm-Bonferroni corrected).

e MRI: magnetic resonance imaging.

f Significant in comparison with GPT-5.2, *P<*.05.

g NM: nuclear medicine.

**Figure 4.**
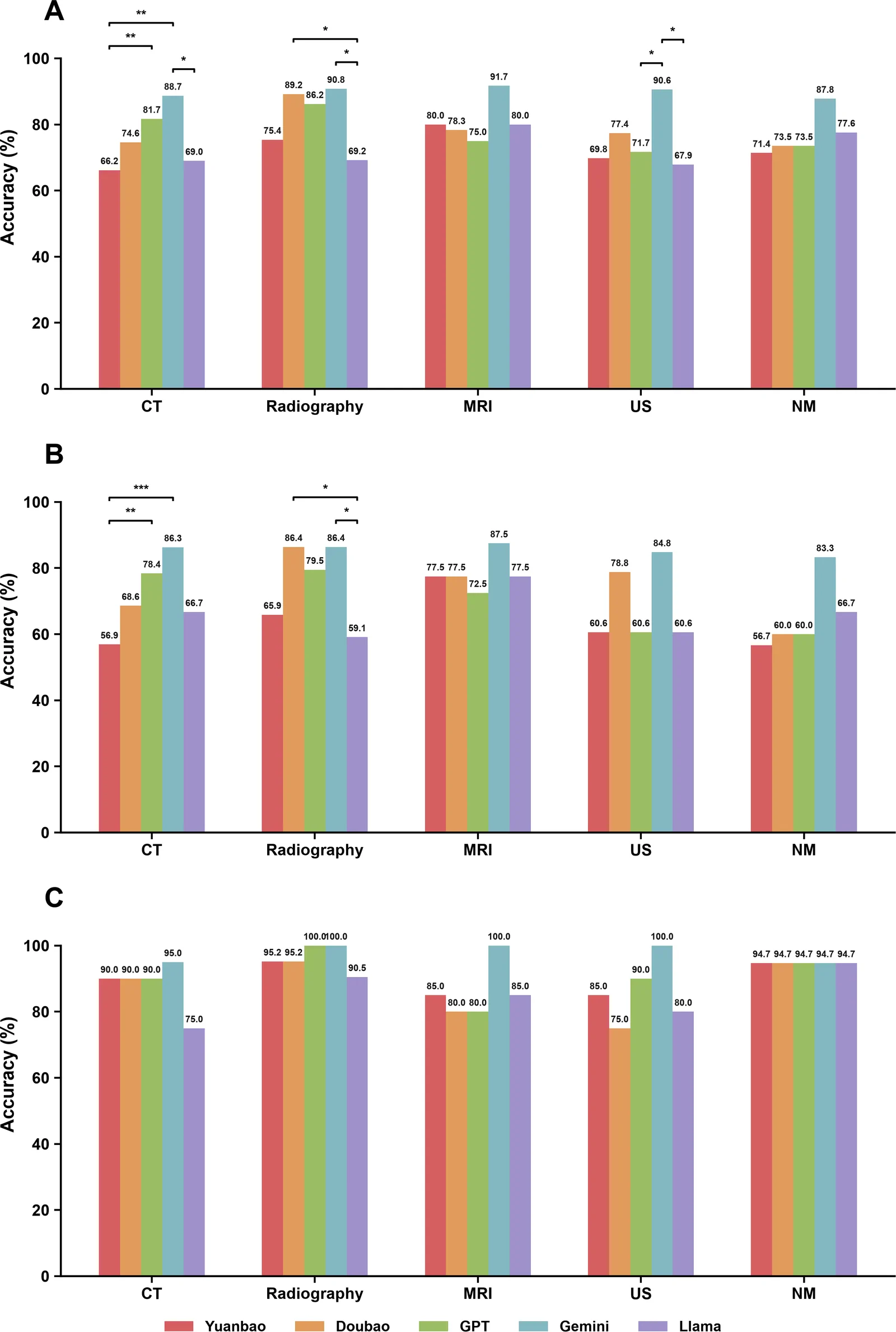
Performance of large language models (LLMs) across different imaging modalities. Bar graphs show the performance of 5 LLMs—Yuanbao, Doubao, GPT (GPT-5.2), Gemini (Gemini 3 Pro), and Llama (Llama 4 Maverick)—in answering medical imaging questions categorized by imaging modality: computed tomography (CT), radiography, magnetic resonance imaging (MRI), ultrasound (US), and nuclear medicine (NM). (A) Performance on the total dataset (combining both image-based and text-based questions). (B) Performance on the subset of image-based questions. (C) Performance on the subset of text-based questions. Significant differences are indicated by brackets and asterisks: *P<.05, **P<.01, ***P<.001.

When restricted to image-based questions, overall differences were observed for CT (*P<*.001), radiography (*P<*.001), and ultrasound (*P=*.02) modalities. Pairwise comparisons showed that for CT questions, Gemini 3 Pro (44/51, 86.3%, 95% CI 74.3%‐93.2%) achieved higher accuracy than Yuanbao (29/51, 56.9%, 95% CI 43.3%‐69.5%; *P<*.001); GPT-5.2 (40/51, 78.4%, 95% CI 65.4%‐87.5%) also outperformed Yuanbao (*P=*.009). For ultrasound questions, although the overall difference was significant (*P=*.02), no pairwise differences remained significant after Holm-Bonferroni correction. For radiography, both Doubao (38/44, 86.4%, 95% CI 73.3%‐93.6%) and Gemini 3 Pro (38/44, 86.4%, 95% CI 73.3%‐93.6%) outperformed Llama 4 Maverick (26/44, 59.1%, 95% CI 44.4%‐72.3%; both *P=*.04). No significant overall differences were detected for MRI or NM (Table 3 and Figure 4B). For text-based questions, no evidence of an overall difference was detected among the 5 models across all 5 imaging modalities (CT, *P*=.28; radiography, *P*=.23; MRI, *P*=.13; ultrasound, *P*=.08; and NM, *P>*.99; Table 3 and Figure 4C).

Regarding intramodel performance, no significant difference in accuracy was detected among the 5 imaging modalities for any of the evaluated models (Yuanbao, *P*=.46; Doubao, *P*=.21; GPT-5.2, *P*=.26; Gemini 3 Pro, *P*=.96; and Llama 4 Maverick, *P*=.45; Figure 5).

**Figure 5.**
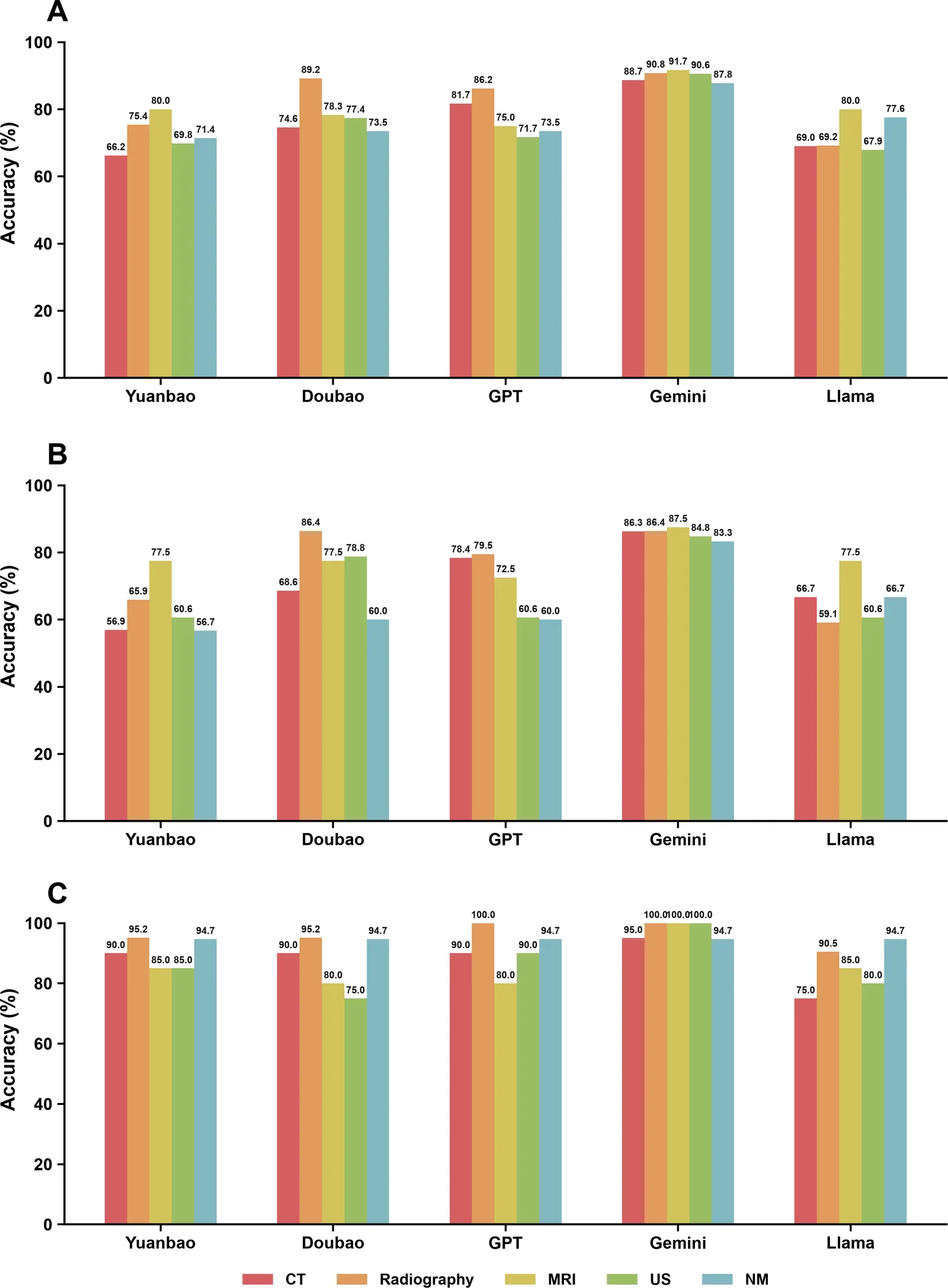
The bar graphs show the intramodel performance stability of 5 multimodal large language models (LLMs)—Yuanbao, Doubao, GPT (GPT-5.2), Gemini (Gemini 3 Pro), and Llama (Llama 4 Maverick)—across 5 imaging modalities: computed tomography (CT), radiography, magnetic resonance imaging (MRI), ultrasound (US), and nuclear medicine (NM). (A) Performance on the total dataset (combining both image-based and text-based questions). (B) Performance on the subset of image-based questions. (C) Performance on the subset of text-based questions. No statistically significant differences in accuracy were observed across these 5 modalities within any of the evaluated models (all *P*>.05).

### Accuracy Analysis Based on the Anatomical System

In the overall dataset stratified by the anatomical system, model accuracy ranged from 65.3% to 95.9%. Overall differences among the 5 models were observed for neurologic (*P=*.04), respiratory (*P<*.001), cardiovascular (*P=*.02), genitourinary (*P=*.04), and musculoskeletal (*P=*.02) systems. No significant overall difference was detected for the digestive system (*P=*.17). Post hoc pairwise comparisons demonstrated that for the respiratory system, Gemini 3 Pro (47/49, 95.9%, 95% CI 86.3%‐98.9%) achieved higher accuracy than Yuanbao (35/49, 71.4%, 95% CI 57.6%‐82.2%; *P=*.02) and Llama 4 Maverick (34/49, 69.4%, 95% CI 55.5%‐80.5%; *P=*.01). Although overall differences were detected for neurologic, cardiovascular, genitourinary, and musculoskeletal systems, no pairwise differences were identified after Holm-Bonferroni correction (Table 4 and Figure 6A).

**Table 4.** Accuracy of large language models stratified by anatomical system^a^.

Anatomical system	N	Yuanbao, accuracy (95% Wilson score CI)	Doubao, accuracy (95% Wilson score CI)	GPT-5.2, accuracy (95% Wilson score CI)	Gemini 3 Pro, accuracy (95% Wilson score CI)	Llama 4 Maverick, accuracy (95% Wilson score CI)	*P* value
Total							
Neurologic	56	73.2 (60.4‐83.0)	76.8 (64.2‐85.9)	78.6 (66.2‐87.3)	91.1 (80.7‐96.1)	73.2 (60.4‐83.0)	.04
Respiratory	49	71.4 (57.6‐82.2)	77.6 (64.1‐87.0)	79.6 (66.4‐88.5)	95.9 (86.3‐98.9)^b^^,^^c^	69.4 (55.5‐80.5)	<.001
Cardiovascular	49	65.3 (51.3‐77.1)	81.6 (68.6‐90.0)	67.3 (53.4‐78.8)	83.7 (71.0‐91.5)	71.4 (57.6‐82.2)	.02
Digestive	61	72.1 (59.8‐81.8)	77.0 (65.1‐85.8)	77.0 (65.1‐85.8)	83.6 (72.4‐90.8)	70.5 (58.1‐80.4)	.17
Genitourinary	39	76.9 (61.7‐87.4)	71.8 (56.2‐83.5)	82.1 (67.3‐91.0)	92.3 (79.7‐97.3)	76.9 (61.7‐87.4)	.04
Musculoskeletal	44	77.3 (63.0‐87.2)	88.6 (76.0‐95.0)	86.4 (73.3‐93.6)	95.5 (84.9‐98.7)	75.0 (60.6‐85.4)	.02
Image-based							
Neurologic	43	69.8 (54.9‐81.4)	74.4 (59.8‐85.1)	76.7 (62.3‐86.8)	90.7 (78.4‐96.3)	72.1 (57.3‐83.3)	.04
Respiratory	30	56.7 (39.2‐72.6)	70.0 (52.1‐83.3)	73.3 (55.6‐85.8)	93.3 (78.7‐98.2)^b^	60.0 (42.3‐75.4)	.002
Cardiovascular	33	57.6 (40.8‐72.8)	78.8 (62.2‐89.3)	57.6 (40.8‐72.8)	75.8 (59.0‐87.2)	63.6 (46.6‐77.8)	.04
Digestive	39	61.5 (45.9‐75.1)	74.4 (58.9‐85.4)	69.2 (53.6‐81.4)	76.9 (61.7‐87.4)	61.5 (45.9‐75.1)	.19
Genitourinary	25	68.0 (48.4‐82.8)	64.0 (44.5‐79.8)	72.0 (52.4‐85.7)	88.0 (70.0‐95.8)	68.0 (48.4‐82.8)	.13
Musculoskeletal	28	67.9 (49.3‐82.1)	85.7 (68.5‐94.3)	82.1 (64.4‐92.1)	92.9 (77.4‐98.0)	71.4 (52.9‐84.7)	.09
Text-based							
Neurologic	13	84.6 (57.8‐95.7)	84.6 (57.8‐95.7)	84.6 (57.8‐95.7)	92.3 (66.7‐98.6)	76.9 (49.7‐91.8)	.86
Respiratory	19	94.7 (75.4‐99.1)	89.5 (68.6‐97.1)	89.5 (68.6‐97.1)	100.0 (83.2‐100.0)	84.2 (62.4‐94.5)	.32
Cardiovascular	16	81.2 (57.0‐93.4)	87.5 (64.0‐96.5)	87.5 (64.0‐96.5)	100.0 (80.6‐100.0)	87.5 (64.0‐96.5)	.41
Digestive	22	90.9 (72.2‐97.5)	81.8 (61.5‐92.7)	90.9 (72.2‐97.5)	95.5 (78.2‐99.2)	86.4 (66.7‐95.3)	.32
Genitourinary	14	92.9 (68.5‐98.7)	85.7 (60.1‐96.0)	100.0 (78.5‐100.0)	100.0 (78.5‐100.0)	92.9 (68.5‐98.7)	.23
Musculoskeletal	16	93.8 (71.7‐98.9)	93.8 (71.7‐98.9)	93.8 (71.7‐98.9)	100.0 (80.6‐100.0)	81.2 (57.0‐93.4)	.09

a Data are presented as accuracy (%) with 95% Wilson score CIs in parentheses. The *P* value in the last column indicates the global difference among the 5 models calculated using the Cochran *Q *test.

b Significant in comparison with Yuanbao, *P<*.05.

c Significant in comparison with Llama 4 Maverick, *P<*.05 (Holm-Bonferroni corrected).

**Figure 6.**
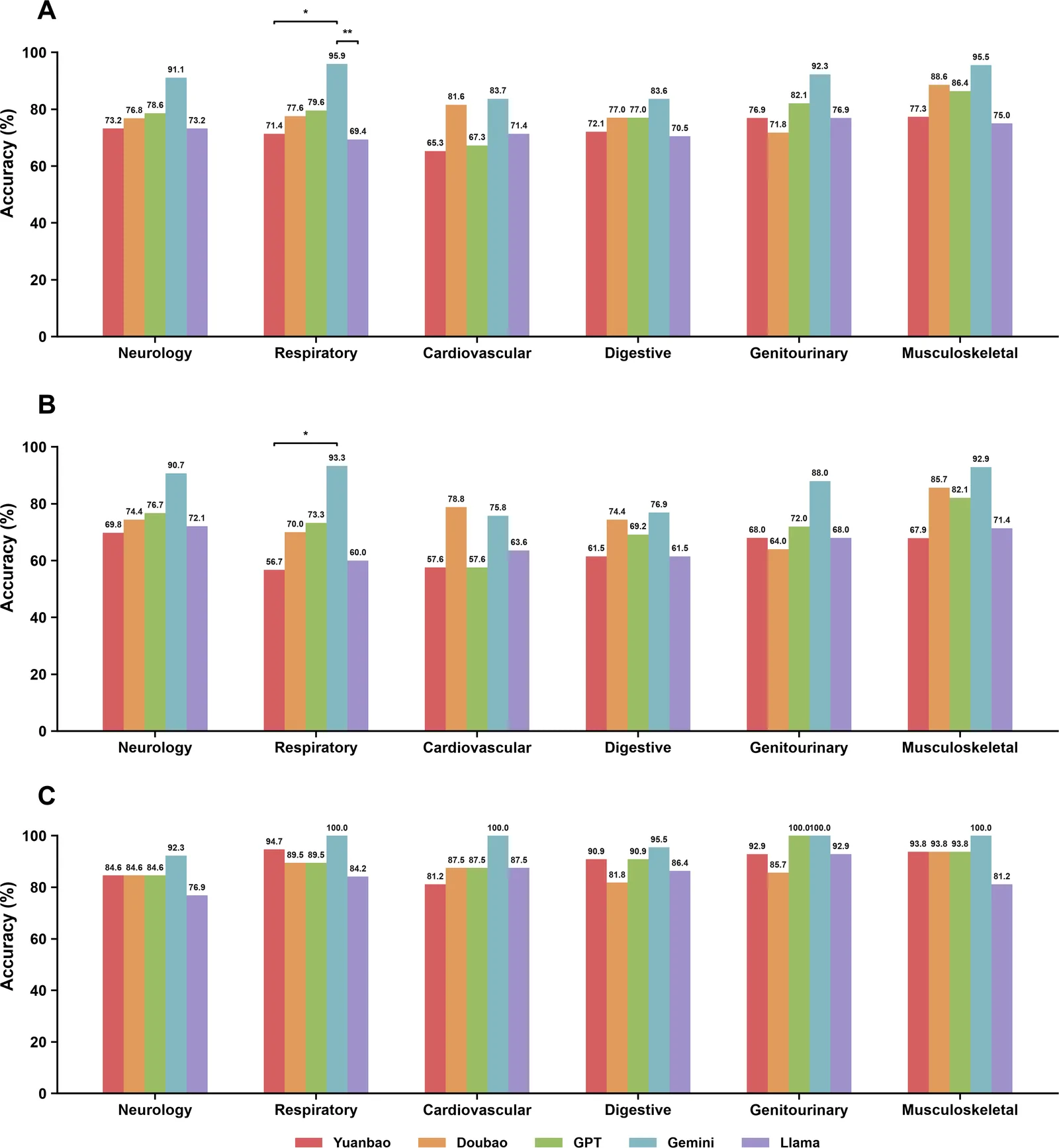
Performance of large language models (LLMs) across different anatomical systems. Bar graphs show the performance of 5 LLMs, including Yuanbao, Doubao, GPT (GPT-5.2), Gemini (Gemini 3 Pro), and Llama (Llama 4 Maverick), in answering medical imaging questions related to 6 anatomical systems: neurologic, respiratory, cardiovascular, digestive, genitourinary, and musculoskeletal. (A) Performance on the total dataset (combining both image-based and text-based questions). (B) Performance on the subset of image-based questions. (C) Performance on the subset of text-based questions. Significant differences are indicated by brackets and asterisks: *P<.05, **P<.01.

When restricted to image-based questions, overall differences were observed for neurologic (*P=*.04), respiratory (*P=*.002), and cardiovascular (*P=*.04) systems. Pairwise comparisons showed that for image-based respiratory questions, Gemini 3 Pro (28/30, 93.3%, 95% CI 78.7%‐98.2%) performed better than Yuanbao (17/30, 56.7%, 95% CI 39.2%‐72.6%; *P=*.03). No significant pairwise differences were detected in the neurologic or cardiovascular systems. For digestive, genitourinary, and musculoskeletal systems, no significant overall differences were detected (Table 4 and Figure 6B). For text-based questions, no overall differences among the 5 models were observed across all 6 anatomical systems (neurologic, *P*=.86; respiratory, *P*=.32; cardiovascular, *P*=.41; digestive, *P*=.32; genitourinary, *P*=.23; musculoskeletal, *P*=.09; Table 4 and Figure 6C).

For intramodel comparisons, no significant difference was detected in accuracy across the 6 anatomical systems for any evaluated model (Yuanbao, *P*=.82; Doubao, *P*=.52; GPT-5.2, *P*=.35; Gemini 3 Pro, *P*=.13; Llama 4 Maverick, *P*=.97; Figure 7).

**Figure 7.**
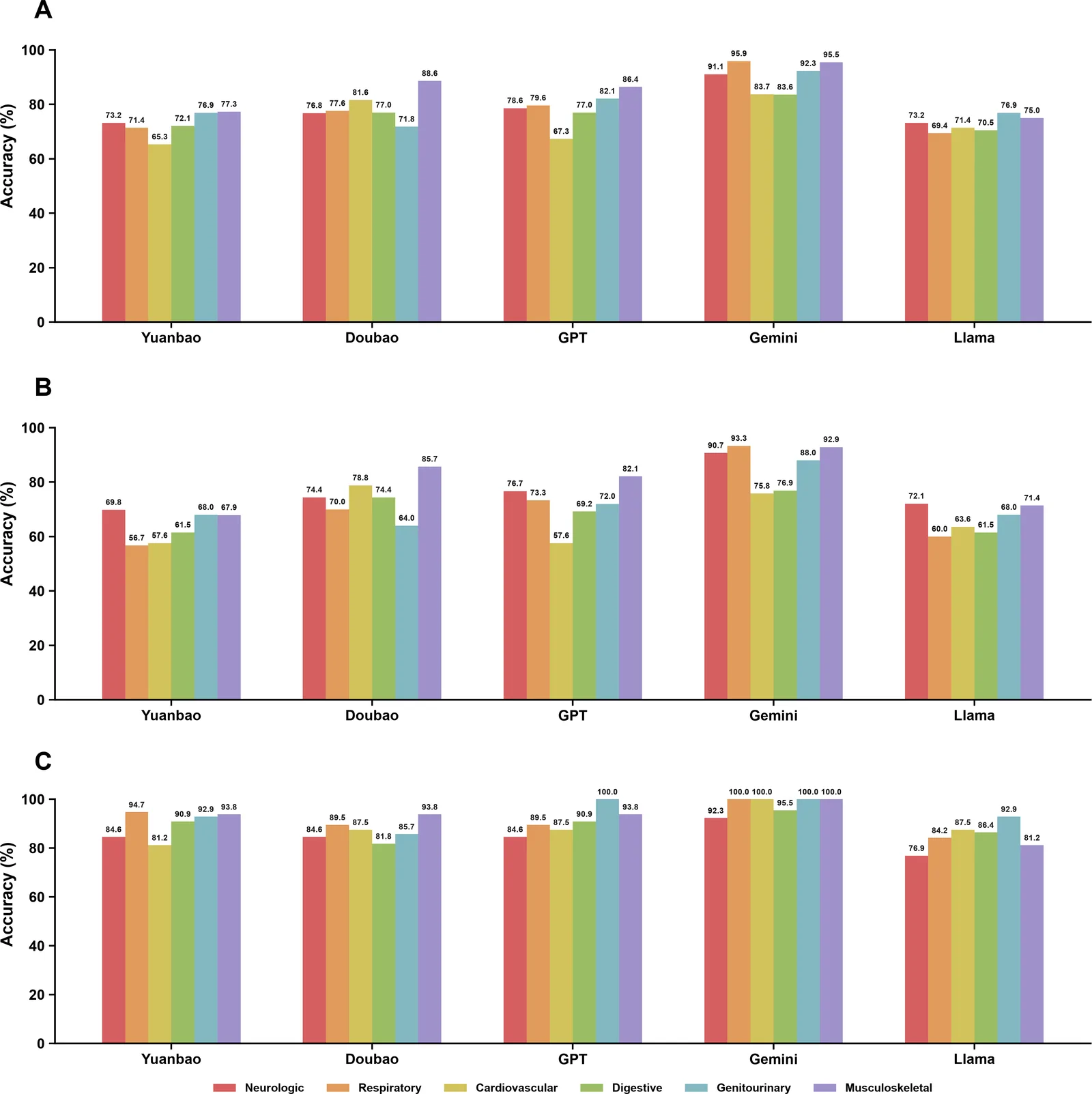
The bar graphs show the intramodel performance stability of 5 multimodal large language models (LLMs)—Yuanbao, Doubao, GPT (GPT-5.2), Gemini (Gemini 3 Pro), and Llama (Llama 4 Maverick)—across 6 anatomical systems (neurologic, respiratory, cardiovascular, digestive, genitourinary, and musculoskeletal). (A) Performance on the total dataset (combining both image-based and text-based questions). (B) Performance on the subset of image-based questions. (C) Performance on the subset of text-based questions. No statistically significant differences in accuracy were observed across the 6 anatomical systems within any of the evaluated models (all *P*>.05).

Given the relatively small number of questions in some modality- and anatomical-system subgroups, these subgroup estimates should be interpreted cautiously in conjunction with their 95% CIs.

### Visual Reasoning Performance on Image-Based Questions

In the evaluation of visual recognition tasks related to imaging modality classification and anatomical structure identification, all 5 models demonstrated high accuracy ranging from 196 of 198 (99.0%, 95% CI 96.4%‐99.7%) to 198 of 198 (100.0%, 95% CI 98.1%‐100.0%). No significant between-model differences were detected for modality recognition or anatomical recognition (*P=*.56 and *P=*.25, respectively).

In contrast to the near-perfect performance in basic visual recognition, model accuracy decreased in more complex visual interpretation tasks, ranging from 68.2% to 88.4% for lesion detection and from 84.8% to 95.5% for lesion localization. For lesion detection, overall differences among models were observed (*P<*.001). Post hoc pairwise comparisons demonstrated that Gemini 3 Pro (175/198, 88.4%, 95% CI 83.2%‐92.1%) achieved higher accuracy than Yuanbao (135/198, 68.2%, 95% CI 61.4%‐74.3%; *P<*.001), GPT-5.2 (152/198, 76.8%, 95% CI 70.4%‐82.1%; *P=*.002), and Llama 4 Maverick (142/198, 71.7%, 95% CI 65.1%‐77.5%; *P<*.001). Additionally, Doubao (159/198, 80.3%, 95% CI 74.2%‐85.2%) outperformed Yuanbao (*P=*.003). No other pairwise differences remained significant after Holm-Bonferroni correction. For lesion localization, overall differences were also observed (*P<*.001). Pairwise comparisons demonstrated that Gemini 3 Pro (189/198, 95.5%, 95% CI 91.6%‐97.6%) outperformed Yuanbao (168/198, 84.8%, 95% CI 79.2%‐89.2%; *P<*.001), Doubao (171/198, 86.4%, 95% CI 80.9%‐90.5%; *P=*.004), and Llama 4 Maverick (172/198, 86.9%, 95% CI 81.5%‐90.9%; *P=*.004). No significant differences were detected among Yuanbao, Doubao, GPT-5.2, and Llama 4 Maverick in lesion localization (Table 5 and Figure 8).

**Table 5.** Performance of large language models (LLMs) across 4 image-interpretation outcomes in image-based questions^a^.

Dimension	Yuanbao, accuracy (95% Wilson score CI)	Doubao, accuracy (95% Wilson score CI)	GPT-5.2, accuracy (95% Wilson score CI)	Gemini 3 Pro, accuracy (95% Wilson score CI)	Llama 4 Maverick, accuracy (95% Wilson score CI)	*P* value
Modality recognition	100.0 (98.1‐100.0)	99.5 (97.2‐99.9)	100.0 (98.1‐100.0)	100.0 (98.1‐100.0)	99.5 (97.2‐99.9)	.56
Anatomical recognition	100.0 (98.1‐100.0)	99.5 (97.2‐99.9)	100.0 (98.1‐100.0)	100.0 (98.1‐100.0)	99.0 (96.4‐99.7)	.25
Lesion detection	68.2 (61.4‐74.3)	80.3 (74.2‐85.2)^b^	76.8 (70.4‐82.1)	88.4 (83.2‐92.1)^b^^,^^c^^,^^d^	71.7 (65.1‐77.5)	<.001
Lesion localization	84.8 (79.2‐89.2)	86.4 (80.9‐90.5)	89.9 (84.9‐93.4)	95.5 (91.6‐97.6)^b,d,e^	86.9 (81.5‐90.9)	<.001

a Data are presented as accuracy (%) with 95% Wilson score CIs in parentheses. The *P* value in the last column indicates the global difference among the 5 models calculated using the Cochran *Q *test.

b Significant in comparison with Yuanbao, *P<*.05.

c Significant in comparison with GPT-5.2, *P<*.05.

d Significant in comparison with Llama 4 Maverick, *P<*.05 (Holm-Bonferroni corrected).

e Significant in comparison with Doubao, *P<*.05.

**Figure 8.**
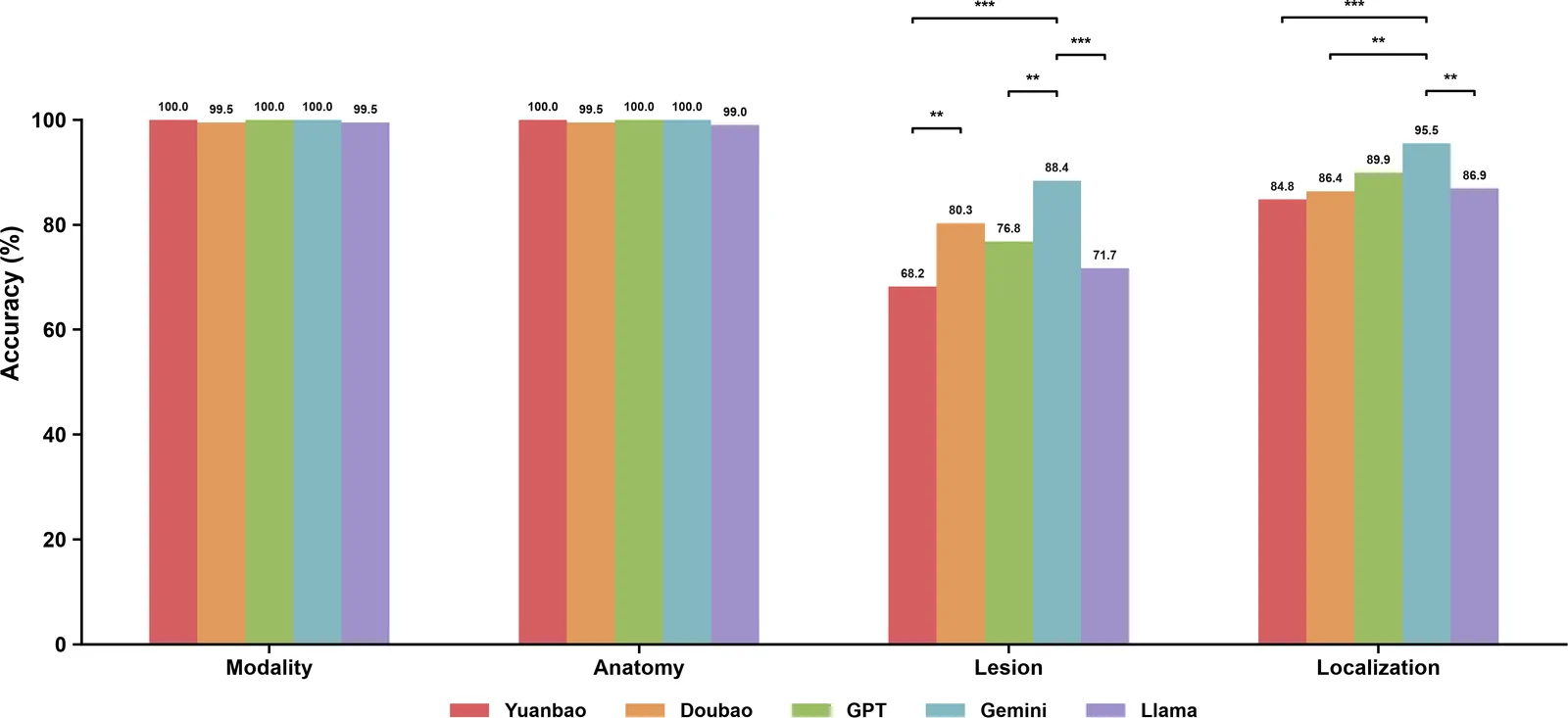
Evaluation of large language model (LLM) performance across 4 key dimensions of image interpretation. Bar graphs show the performance of 5 LLMs, including Yuanbao, Doubao, GPT (GPT-5.2), Gemini (Gemini 3 Pro), and Llama (Llama 4 Maverick), in identifying specific imaging features: imaging modality classification (modality), anatomical structure recognition (anatomy), lesion detection (lesion), and lesion localization (localization). The comparison highlights model-specific strengths and weaknesses in extracting fine-grained medical information from images. Significant differences are indicated by brackets and asterisks: *P<.05, **P<.01, ***P<.001.

Interrater agreement was evaluated across 4 diagnostic dimensions: modality, anatomy, lesion, and localization. Observed agreement was very high, ranging from 97.5% to 99.5%. Because several dimensions exhibited highly skewed marginal distributions, PABAK was reported alongside the standard Cohen κ. PABAK ranged from 0.95 to 0.99, indicating almost perfect agreement across all dimensions (Table S5 in Multimedia Appendix 1).

## Discussion

### Principal Findings

This cross-sectional benchmark study evaluated the performance of 5 multimodal LLMs in answering medical imaging MCQs covering bilingual contexts, dual question types (image and text), multiple imaging modalities, and various anatomical systems. Our primary findings were as follows: first, Gemini 3 Pro achieved the highest overall accuracy; second, all evaluated models performed better on text-only questions than on image-based questions; finally, an in-depth analysis of visual dimensions revealed that although multimodal LLMs achieved near-perfect accuracy in basic visual recognition (eg, recognizing modality and anatomy), their performance declined and varied greatly among models in lesion detection and localization tasks involving more fine-grained lesion-level interpretation.

### Comparison With Prior Work

Regarding overall performance and language stratification, Gemini 3 Pro demonstrated a clear advantage, while no significant overall differences were detected among Doubao, GPT-5.2, Yuanbao, and Llama 4 Maverick. The optimal comprehensive performance of Gemini 3 Pro may be related to its underlying native multimodal architecture [8]. Unlike traditional spliced visual models, Gemini natively integrates text and image data from the ground up. This architecture may facilitate information retention and reduce cross-modal loss when extracting complex medical imaging features and aligning them with clinical histories. Furthermore, its advantage in long-context reasoning may support its global logical deduction capabilities when handling complex medical MCQs with long stems [21]. In the subgroup analysis of language preference, we observed that GPT-5.2, Llama 4 Maverick, and Yuanbao achieved higher accuracy on English questions than on Chinese questions. This performance discrepancy is likely related to the composition of the corpora they were exposed to during pretraining. As noted by Bhayana [1], the pretraining data of current mainstream LLMs primarily rely on massive amounts of unstructured text from the internet, where high-quality medical literature, open-source imaging databases, and medical guidelines are overwhelmingly dominated by the English language. Notably, as a representative regional model, Doubao demonstrated an overall accuracy comparable to that of GPT-5.2 and achieved performance with no evidence of a difference from Gemini 3 Pro on Chinese-language questions. This pattern may be consistent with potential benefits of alignment or fine-tuning with high-quality localized medical corpora for language-specific clinical tasks [9]. The inclusion of Llama 4 Maverick also provides an open-weight reproducibility reference alongside the proprietary web-based systems. Previous medical studies have demonstrated the competitive performance of Llama-family models: Adams et al [22] reported that Llama 3 70B performed comparably to leading proprietary models on radiology board-style questions, while Fast et al [23] found Llama 3 70B to be among the highest-performing models in a medical benchmark spanning 13 specialties. These findings support our selection of Llama 4 Maverick as a representative open-weight comparator; its fixed model revision and documented inference configuration further enable evaluation under reproducible model conditions.

An important finding of this study is that the accuracy of all evaluated models on image-based questions was lower than that on text-only questions. This result is highly consistent with several recent studies evaluating the radiological capabilities of large models. For example, Atakır et al [5] found in their evaluation of ChatGPT-4o that its accuracy was highest under conditions with rich text input, while its performance was limited in pure image tasks. Hayden et al [6] also observed a similar “text-outperforms-image” phenomenon when evaluating the American College of Radiology question bank. A possible reason for this gap is that the logical reasoning capabilities of current LLMs in natural language processing are highly mature; however, within a multimodal architecture, the mapping process of aligning complex medical image pixel features to the text space may remain challenging. Recent studies indicate that limited sensitivity to subtle grayscale contrast may lead visual encoders to lose critical spatial and contextual information when extracting fine-grained features, such as lesion margins [3,24]. Prior studies suggest that models may derive substantial diagnostic information from textual clinical details, whereas performance can decline when visual information must be interpreted more directly [5]. The performance of Yuanbao in this evaluation illustrates the observed gap between strong text-oriented reasoning and more variable image-related performance in some multimodal systems. Although Yuanbao was evaluated with the DeepSeek-R1 option selected, renowned for its exceptional logical reasoning capabilities, its performance on image-based questions (particularly lesion detection) was substantially constrained. This may reflect differences in visual processing or cross-modal alignment, although the underlying mechanism cannot be determined from the present benchmark. More generally, limitations in visual feature extraction could constrain downstream reasoning when multimodal systems process complex radiological images [25].

In the evaluations across different imaging modalities and anatomical systems, our intramodel analysis showed that all models maintained stable accuracy when dealing with different modalities (CT, radiography, MRI; Yuanbao, *P*=.46; Doubao, *P*=.21; GPT-5.2, *P*=.26; Gemini 3 Pro, *P*=.96; and Llama 4 Maverick, *P*=.45) and systems (Yuanbao, *P*=.82; Doubao, *P*=.52; GPT-5.2, *P*=.35; Gemini 3 Pro, *P*=.13; and Llama 4 Maverick, *P*=.97). Given the relatively small sizes of some modality and anatomical-system subgroups, these findings should be interpreted cautiously and do not establish equivalent performance across subgroups. However, in intermodel comparisons, significant pairwise differences involving Gemini 3 Pro were observed in CT, radiography, ultrasound, and the respiratory system. This performance discrepancy may reflect the different emphases of these models in their multimodal pretraining strategies and specialty data coverage. First, at the modality level, CT requires interpretation of complex cross-sectional spatial anatomy, radiography involves superimposed projectional structures, while ultrasound demands the recognition of nonstandardized planes and high-noise acoustic textures. Currently, most multimodal LLMs face bottlenecks in aligning such modality-specific physical features [24]. The observed differences across these modalities may reflect differences in visual training data or multimodal alignment. Second, at the anatomical system level, the respiratory system was the only subgroup in which significant pairwise differences remained after multiple-comparison corrections. This system-specific difference is consistent with the view of Mukherjee et al [26]: when faced with medical imaging characterized by high professional barriers, the reasoning ceiling of a model is deeply constrained by the distribution of its underlying specialty training data. This difference may also stem from variations in model-specific training and alignment strategies [27].

The stratified analysis of higher-order visual dimensions for image-based questions further characterizes the specific limitations of multimodal LLMs in radiological visual interpretation. In basic macroscopic visual tasks, such as modality recognition and anatomical recognition, all models reached a ceiling effect (approaching 100%). However, when progressing to higher-order tasks such as lesion detection and localization, model performance showed noticeable divergence, and overall accuracy dropped. This suggests a limitation in fine-grained image interpretation, although the underlying architectural mechanisms were not directly evaluated in this study. They can easily identify that an image is a head CT while showing weaker performance in tasks requiring more fine-grained local lesion interpretation. In real-world radiological diagnosis, identifying lesion margins, subtle density changes, and precise spatial adjacency relationships is crucial. Limitations in fine-grained visual perception may lead large models to produce visual hallucinations or miss diagnoses in complex medical images [1]. Unlike previous studies that treated visual interpretation largely as a “black box” by focusing solely on overall accuracy, our fine-grained deconstruction uniquely illuminates the specific sources of these errors. Consequently, this finding suggests that future model development may benefit from greater attention to local feature perception.

### Limitations

This study had several limitations. First, although we conducted a systematic evaluation of the models using a standardized benchmark, the sample size was relatively limited (N=298), and some modality- and anatomical-system subgroups contained relatively few questions, which may limit the precision and generalizability of subgroup estimates. Second, because the test questions originated from past examination banks and publicly available educational sources, prior exposure or benchmark contamination cannot be excluded, and differential exposure across models may have influenced cross-model comparisons. Targeted spot-checks confirmed that some public-source items were searchable online verbatim or near-verbatim. Third, because image-based questions also contained textual information and no formal input-ablation analysis was performed, the independent contribution of visual input could not be isolated. Fourth, this experiment used standardized zero-shot prompting and did not evaluate performance under optimized prompting strategies. Fifth, LLMs undergo extremely rapid iterative updates. The 4 proprietary web-based systems remain subject to vendor updates, limiting independent reproducibility. Although the fixed Llama 4 Maverick revision provides an open-weight reproducibility anchor, it was evaluated later than the 4 proprietary systems, and the findings should still be regarded as a time-stamped benchmark.

### Conclusions

Multimodal LLMs, represented by Gemini 3 Pro, demonstrated outstanding overall performance and broad cross-modal adaptability in medical imaging MCQs, highlighting the potential of multimodal LLMs as tools for clinical education and decision support. However, performance varied across languages, question types, and specific image-interpretation tasks, with lower and more variable performance in lesion detection and localization than in modality and anatomical recognition. Future research and model development should focus on further improving fine-grained lesion-level image interpretation and validating these systems on independent clinical imaging datasets before clinical implementation.

## Supplementary material

10.2196/98849 Multimedia Appendix 1 Question provenance, configurations and access conditions of proprietary and open-weights models, pairwise comparisons of final-answer accuracy, and interrater agreement for image-interpretation outcomes.

## Abbreviations

CT: computed tomography
LLM: large language model
MCQ: multiple-choice question
MRI: magnetic resonance imaging
NM: nuclear medicine
PABAK: prevalence-adjusted and bias-adjusted kappa
