# Supplementary material for: Diagnostic Accuracy and Fine-Grained Image-Interpretation Performance of Multimodal Large Language Models on Bilingual Radiology Questions: Cross-Sectional Benchmark Study
Source: J Med Internet Res. 2026 Nov 11;28:e98849. doi: 10.2196/98849 (PMC13754182; doi:10.2196/98849)
Supplement: Supplementary file 1 [file jmir-v28-e98849-s001.docx]

| **Table S1. Sources and Publication Periods of Questions Included in the Benchmark** | | | | |  |
| --- | --- | --- | --- | --- | --- |
| Source | N | Included Question IDs | Language | Publication Period | |
| Chinese Senior Health Professional Qualification Examination question banks | 160 | 1–50, 101–150, 239–298 | Chinese | 2010-2023 | |
| New England Journal of Medicine Image Challenge | 71 | 51–83, 91–94, 201–234 | English | 2006-2025 | |
| BMJ Emergency Medicine Journal Image Challenge | 7 | 84–90 | English | 2022-2025 | |
| Asian Nuclear Medicine Board sample questions | 6 | 95–100 | English | 2018-2024 | |
| Radiopaedia MCQs | 54 | 151–200, 235–238 | English | 2016-2025 | |

**Note.** N indicates the number of included questions. Question IDs correspond to the internal numbering used in the benchmark dataset. Publication periods represent the identifiable publication year or available time range for each source. MCQ = multiple-choice question

**Table S2. Configuration and Access Conditions of Four Proprietary Multimodal Large Language Models Evaluated via Official Web Interfaces**

| Characteristic | | GPT-5.2 | Gemini 3 Pro | Doubao | Yuanbao | |
| --- | --- | --- | --- | --- | --- | --- |
| Developer | | OpenAI | Google | ByteDance | Tencent | |
| Exact model designation (user-facing label) | | GPT-5.2 | Gemini 3 Pro | Doubao | Tencent Yuanbao with DeepSeek-R1 option selected | |
| Access platform | | Official ChatGPT consumer-facing web interface; no API | Official Gemini consumer-facing web interface; no API | Official Doubao consumer-facing web interface; no API | Official Tencent Yuanbao consumer-facing web interface; no API | |
| Evaluation period | | December 14, 2025–January 31, 2026 | December 14, 2025–January 31, 2026 | December 14, 2025–January 31, 2026 | December 14, 2025–January 31, 2026 | |
| Geographic region | | Mainland China | Mainland China | Mainland China | Mainland China | |
| Subscription tier and reasoning setting | | Plus subscription; Auto mode selected | Pro subscription; Pro mode selected | Free account; Deep Thinking mode selected | Free account; DeepSeek-R1 with Deep Thinking mode selected | |
| Internet/search status | | No web search was intentionally invoked; | No web search was intentionally invoked; | No web search was intentionally invoked; | No web search was intentionally invoked; | |
| Image-upload conditions | | Identical source images were used across all four systems; no image preprocessing was performed. | Identical source images were used across all four systems; no image preprocessing was performed. | Identical source images were used across all four systems; no image preprocessing was performed. | Identical source images were used across all four systems; no image preprocessing was performed. | |
| Prompting strategy | | Standardized zero-shot prompting | Standardized zero-shot prompting | Standardized zero-shot prompting | Standardized zero-shot prompting | |
| Conversation procedure | | A new conversation was initiated for each question | A new conversation was initiated for each question | A new conversation was initiated for each question | A new conversation was initiated for each question | |
| Number of attempts per question | | One attempt per question; no regeneration. | One attempt per question; no regeneration. | One attempt per question; no regeneration. | One attempt per question; no regeneration. | |
|  | **Note.** Model names refer to the user-facing labels displayed during the evaluation period. Backend model snapshot identifiers and inference parameters, including temperature, top-p, and random seed, were not exposed through the proprietary consumer-facing web interfaces and therefore could not be obtained. API = application programming interface | | | | |  |

**Table S3. Configuration of the Open-Weight Llama 4 Maverick Model**

| Configuration item | Setting |
| --- | --- |
| Developer | Meta |
| Model | Llama 4 Maverick 17B 128E Instruct |
| Model revision | rv_CcoU69kmhykJ1cXMkQ5wk |
| Model type | Open-weight, natively multimodal large language model |
| Architecture | Mixture-of-experts; 17B activated parameters, 128 experts |
| Supported input modality | Text and image |
| Official release date | Apr-25 |
| Reported pretraining-data cutoff | Aug-24 |
| Inference precision | NVFP4 |
| Hardware | 4 × NVIDIA B200 GPUs |
| GPU memory | 180 GB per GPU |
| Evaluation period | Sep-26 |
| API interface | Together AI OpenAI-compatible Chat Completions API |
| Temperature | 0 |
| Maximum output tokens | 2000 |
| Conversation history | None; each question was submitted as an independent request |
| Image transmission | Base64-encoded image data URL |
| Image integrity record | SHA-256 hash recorded for each submitted image |
| Web search | Not used |
| Response regeneration | No regeneration after a successful response |

**Note.** NVFP4 = NVIDIA 4-bit floating point; GPU = graphics processing unit; API = application programming interface.SHA-256 = Secure Hash Algorithm 256-bit.

**Table S4. Pairwise Differences in Final-Answer Accuracy among the Five Evaluated Multimodal Large Language Model Systems**

| Comparison | Accuracy Difference, Percentage Points | 95% CI for Accuracy Difference, Percentage Points | Raw McNemar P Value | Holm-Adjusted P Value |  |
| --- | --- | --- | --- | --- | --- |
| Gemini 3 Pro vs GPT-5.2 | 11.74 | 7.03 to 16.60 | <.001 | <.001 |  |
| Gemini 3 Pro vs Doubao | 11.07 | 5.78 to 16.41 | <.001 | <.001 |  |
| Gemini 3 Pro vs Tencent Yuanbao | 17.45 | 12.19 to 22.76 | <.001 | <.001 |  |
| Gemini 3 Pro vs Llama 4 Maverick | 17.45 | 12.10 to 22.84 | <.001 | <.001 |  |
| GPT-5.2 vs Doubao | -0.67 | -6.22 to 4.88 | .905 | 1.000 |  |
| GPT-5.2 vs Tencent Yuanbao | 5.70 | 0.39 to 11.01 | .046 | .145 |  |
| GPT-5.2 vs Llama 4 Maverick | 5.70 | 0.64 to 10.76 | .036 | .145 |  |
| Doubao vs Tencent Yuanbao | 6.38 | 1.24 to 11.51 | .020 | .102 |  |
| Doubao vs Llama 4 Maverick | 6.38 | 1.41 to 11.34 | .016 | .098 |  |
| Tencent Yuanbao vs Llama 4 Maverick | 0.00 | -5.22 to 5.22 | 1.000 | 1.000 |  |
| **Note.** Overall Cochran Q test: Q=56.24, df=4, P<.001. Accuracy difference was calculated as the accuracy of the first-listed model minus that of the second-listed model and is expressed in percentage points; positive values favor the first-listed model. The 95% confidence intervals for paired accuracy differences were calculated using Newcombe's score-based method for paired proportions (method 10). Two-sided exact McNemar tests were used for pairwise comparisons, and P values were adjusted across the 10 pairwise comparisons using the Holm-Bonferroni procedure. Accuracy differences and confidence interval limits are reported to two decimal places; P values are reported to three decimal places, with values <.001 shown as <.001. CI = confidence interval; df = degrees of freedom. | | | | | |

**Table S5. Inter-rater Agreement for Four Image-Interpretation Outcomes.**

| Dimension | Observed agreement, n/N (%) | Cohen’s κ (95% CI) | PABAK (95% CI) |
| --- | --- | --- | --- |
| Modality | 198/200 (99.00) | 0.50 (-0.10-1.00) | 0.98 (0.93-1.00) |
| Anatomy | 199/200 (99.50) | 0.00 (-0.00-0.00) | 0.99 (0.94-1.00) |
| Lesion | 195/200 (97.50) | 0.93 (0.87-0.99) | 0.95 (0.89-0.98) |
| Localization | 197/200 (98.50) | 0.86 (0.71-1.00) | 0.97 (0.91-0.99) |

Note—Standard Cohen's κ is reported alongside PABAK to illustrate the prevalence effect (Kappa paradox) caused by the highly skewed marginal distribution. In particular, the Anatomy dimension yielded a Cohen's κ of 0.00 despite an observed agreement of 99.5%; this near-zero κ is attributable to the prevalence effect rather than to unreliable scoring, and the PABAK value of 0.99 more faithfully represents the underlying agreement. PABAK = prevalence- and bias-adjusted kappa; CI = confidence interval.
